# 化疗对肺癌患者生活质量的影响

**DOI:** 10.3779/j.issn.1009-3419.2013.12.07

**Published:** 2013-12-20

**Authors:** 旭 刘, 燕 王, 淑芳 李, 士珍 辛, 建存 曹

**Affiliations:** 1 300070 天津，天津医科大学研究生院 Graduate School of Tianjin Medical University, Tianjin 300070, China; 2 300052 天津，天津医科大学总医院肿瘤科 Department of Medical Oncology, Tianjin Medical University General Hospital, Tianjin 300052, China; 3 300380 天津，天津市西青医院呼吸科 Department of Respiration, Tianjin Xiqing Hospital, Tianjin 300380, China; 4 300350 天津，天津海河医院呼吸科 Department of Respiration, Tianjin Haihe Hospital, Tianjin 300350, China

**Keywords:** 肺肿瘤, 化疗, 生活质量, Lung neoplasms, Chemotherapy, Quality of life

## Abstract

**背景与目的:**

随着现代医学的发展，不仅要求医护人员要消除患者病痛和心理障碍，还要提高患者生活质量。本研究探讨化疗对肺癌患者生活质量的影响及其影响因素。

**方法:**

随机抽取住院化疗的肺癌患者61例，分别于化疗前、化疗2周期后1周内、化疗4周期后1周内评估临床疗效，并进行肺癌患者生活质量量表（EORTC QLQ-30）问卷调查。

**结果:**

化疗2周期后化疗有效率40%，患者社会功能下降，恶心、呕吐、失眠、厌食症状加重（*P* < 0.05）。化疗4周期后化疗有效率23%，患者化疗后躯体、角色、情绪、社会功能降低，恶心呕吐、食欲减退、失眠症状加重，经济困难较化疗前加重（*P* < 0.05）。生活质量影响因素分析：女性较男性易疲倦、食欲减退（*P* < 0.05）。文化程度低者较文化程度高者易失眠、疲倦（*P* < 0.05）。老年患者较中年人更易出现疲倦、食欲减退、恶心呕吐（*P* < 0.05）。按体重指数分为消瘦、正常、超重三组，三组比较生活质量无明显统计学差异（*P*>0.05）。按血浆白蛋白水平分为低白蛋白组及正常白蛋白组，两组比较生活质量无明显统计学差异（*P*>0.05）。但前组症状及经济困难得分较高，后组功能领域及总体健康状况得分较高。

**结论:**

化疗可使肺癌患者肿瘤减小，临床症状缓解，但化疗后患者易疲劳、纳差、恶心呕吐加重，患者情绪较差，生活质量降低，应注重患者化疗不良反应并及时予处理，加强对患者的心理疏导，增加患者的营养，提高患者的生活质量。

## Abstract (en)

**Background and objective:**

With development of modern medicine, eliminating patients' pain and mental disorder and improving the quality of life has become an important problem in patients with cancer. The aim of this study is to observe the impact of chemotherapy on quality of life and influencing factor of quality of life among lung cancer patients.

**Methods:**

Sixty-one lung cancer patients were assessed with clinical outcomes and the EORTC QLQ-C30 questionnaires before chemotherapy, one week after 2 cycles of chemotherapy, one week after 4 cycles of chemotherapy.

**Results:**

After 2 cycles of chemotherapy, effective rate was 40.0%. Social function decreased. Nause and vomiting, insomnia and appetite loss deteriorated (*P* < 0.05). After 4 cycles of chemotherapy, effective rate was 23%. But after chemotherapy, physical, role, emotional, social function decreased; Appetite loss, nausea and vomiting, insomnia deteriorated, economics became difficult to some patients (*P* < 0.05). In our study, we find female patients had more fatigue and appetite loss than male patients (*P* < 0.05). Patients of low level of education had more fatigue and insomnia than patients of high level of education (*P* < 0.05). Elder patients had more fatigue, appetite loss, nausea and vomiting than middle-aged patients (*P* < 0.05). Grouped according to body mass index, the difference wasn't statistically significant (*P*>0.05). Grouped according to the plasma albumin level, the difference wasn't statistically significant (*P*>0.05). In hypoproteinemia group, symptoms and economic difficulties score was higher; High protein group, function and general health scores was higher.

**Conclusion:**

After the chemotherapy, patients' the lesion of cancer became smaller and clinical symptoms relieved, but some patients, fatigue, appetite loss, nausea and vomiting symptom deteriorated, emotion became bad. Quality of life of lung cancer patients decrease. We should pay more attention to the adverse reactions of chemotherapy and cope with them, give positive psychological intervention and improve patients, nutrition to improve the quality of life.

原发性支气管肺癌（简称肺癌）是全球最主要的癌症，最新资料^[1]^显示，到2015年，我国将成为世界第一肺癌大国。虽然多学科不断进步，但肺癌仍是高死亡率，不少患者初诊时已经失去了手术的机会^[2]^，化疗是晚期肺癌最常用的治疗手段之一。治疗时不仅要注重患者的生存率，还要重视其生活质量^[3]^。“让癌症患者生存得更长，生活得更好”已经成为当今肿瘤治疗的新理念^[4]^。本研究旨在通过化疗前后患者生活质量的变化及影响因素的分析，为医务人员更好地改善患者的生活质量提供依据。

## 1 资料与方法

### 1.1 临床资料

2012年4月-2013年4月随机选取天津医科大学总医院肿瘤科收治的已确诊肺癌患者61例。纳入标准：①首次病理确诊的原发性支气管肺癌并未进行过化疗的患者；②根据2009年美国国立综合癌症网络（National Comprehensive Cancer Network, NCCN）肺癌国际分期标准为Ⅲ期-Ⅳ期；③体力评分（Karnofsky' s index of performance status, KPS）评分≥60分；④预期生存期 > 3个月；⑤愿意接受问卷调查。排除标准：①既往精神病史；②经解释后仍不能理解问卷条目者；③既往接受抗焦虑或抑郁治疗者；④既往酒精或药物依赖史。

### 1.2 方法

化疗前记录患者的一般资料，包括患者的性别、年龄、身体质量指数（body mass index, BMI）、血浆白蛋白数值、KPS评分、病理类型、肺癌分期、化疗方案、文化程度、有无基础疾病等。分别于化疗前、化疗2周期后1星期内、化疗4周期后1星期内应用欧洲癌症研究与治疗组织开发的生活质量核心量表（European Organization for Research and Treatment EORTCQLQ-30）^[5]^对患者进行问卷调查。

### 1.3 资料收集

研究者对入选患者进行跟踪调查并进行临床疗效的评价。按照RECIST（1.1版）标准^[6]^，疗效评价首先对目标病灶及非目标病灶分别评价，然后综合目标病灶及非目标病灶评价结果进行总评价。目标病灶评价：①完全缓解（complete response, CR）：所有已知病灶消失，无新病灶出现，且肿瘤标志物正常，至少维持4周；②部分缓解（partial response, PR）：肿瘤基线病灶长径总和缩小30%并保持4周以上；③病灶稳定（stable disease, SD）：基线病灶长径总和有缩小但未达PR或增加但未到PD；④疾病进展（progressive disease, PD）：基线病灶长径总和增加≥20%，及其绝对值增加至少5 mm，或出现新病灶。非目标病灶评价：CR：所有非目标病灶消失和肿瘤标志物水平正常；PD：出现一个或多个新病灶和（或）存在非目标病灶进展；SD：一个或多个非目标病灶和（或）肿瘤标志物高于正常持续存在。

总有效率为PR+CR人数之和所占治疗观察人数的百分率。同时对患者行EORTC QLQ-C30问卷调查。EORTC QLQ-C30量表：对于功能领域和总体健康状况领域得分（标化分）越高说明功能状况和生命质量越好，对于症状领域得分越高表明症状或问题越多。万崇华等^[7]^通过对226例恶性肿瘤患者进行的生活质量测定对量表进行评价，说明EORTC QLQ-C30中文版具有良好的信度、效度和反应度，可用于中国癌症患者的生活质量测定。

### 1.4 统计分析

应用SPSS 16.0统计软件，对化疗不同阶段生活质量与患者性别、年龄、文化程度、生活质量各条目间关系进行分析。得分如果符合正态分布，用Mean±SD表示，采用*t*检验、方差分析和SNK两两比较法；如不符合正态分布，用中位数表示，采用*Kruskal-Wallis*秩和检验，*P* < 0.05为差异有统计学意义。

## 2 结果

### 2.1 一般资料

共向61例患者行问卷调查，有效问卷（完成3次问卷者）52例。8例因肿瘤进展放弃继续化疗，1例因急性心肌梗塞放弃化疗。52例患者中，男37例，女15例；年龄50岁-76岁，中位年龄61岁；鳞癌17例，腺癌18例，小细胞癌13例，低分化癌4例；Ⅲ期22例，Ⅳ期30例；有基础疾病者22例（包括高血压、冠心病、慢性支气管炎、支气管哮喘、脑出血、糖尿病等），无基础疾病者30例。化疗方案：非小细胞肺癌一线化疗予含铂两药方案（铂类+吉西他滨、长春瑞滨、紫杉醇、多西他赛等），小细胞肺癌一线化疗予依托泊甙或替尼泊苷+铂类方案，疾病进展给予相应的二线方案，疾病无进展者均继续原方案化疗，化疗每2周期后进行病情评估。

### 2.2 临床疗效

化疗2周期后，CR 0例，PR 21例，SD 12例，PD 19例，有效率为40%；化疗4周期后，CR 0例，PR 12例，SD 10例，PD 30例，有效率为23%。

### 2.3 化疗前后生活质量的变化

肺癌患者化疗前后EORTCQLQ-30生活质量各领域得分变化见表 1。从表中可以看出，患者躯体功能、角色功能、情绪功能、总健康状况化疗4周期后较化疗2周期后及化疗前均明显下降（*P* < 0.05）。认知功能化疗前后无变化（*P*>0.05）。社会功能化疗4周期后较化疗2周期后及化疗前明显下降（*P*＜0.05），化疗2周期后评估较化疗前也下降（*P* < 0.05）。恶心与呕吐症状得分随化疗而升高，恶心与呕吐化疗4周期后较化疗2周期后及化疗前加重，化疗2周期后评估较化疗前也加重（*P* < 0.05）。疼痛症状化疗4周期后较化疗前得分明显增加，疼痛症状加重（*P* < 0.05）。失眠症状：化疗4周期后、化疗2周期后均较化疗前明显加重（*P* < 0.05）。食欲减退症状3次比较均不同，食欲减退症状得分逐渐升高，食欲减退症状逐渐加重（*P* < 0.05）。呼吸困难、疲劳、便秘、腹泻症状3次比较无统计学差异（*P*>0.05），经济困难方面化疗4周期后较化疗2周期后及化疗前得分明显升高（*P* < 0.05）。

**1:** 肺癌患者化疗前后不同时期EORTC-C30各领域得分比较（Mean±SD） Compare of the scores for EORTC QLQ-C30 in different periods of chemotherapy (Mean±SD)

	Baseline	After 2 cycles	After 4 cycles	*F*	*P*
Physical	79.964±11.145	74.390±10.320	67.809±14.812^△※^	5.477	0.005
Role	65.064±23.637	61.858±19.064	51.921±20.270^△※^	5.495	0.005
Emotional	63.603±25.890	62.173±20.717	49.648±18.230^△※^	6.420	0.002
Cognitive	64.721±28.890	67.931±21.592	67.623±18.201	0.299	0.742
Social	63.131±23.432	55.452±77.391^*^	42.622±12.553^△※^	16.611	0.000
Fatigue	49.731±25.152	48.543±21.901	54.722±20.042	1.107	0.333
Pain	25.642±24.790	30.772±21.990	37.182±23.241^※^	3.184	0.044
Nause and vomiting	20.832±20.012	29.704±20.841^*^	37.513±20.051^△※^	8.459	0.000
Dyspnoea	49.351±33.990	37.163±25.281	41.022±24.371	2.535	0.083
Insomnia	32.042±31.641	45.512±26.441^*^	46.153±24.851^※^	4.270	0.160
Appetite loss	33.974±30.610	46.793±28.221^*^	64.754±27.561^△※^	14.96	0.000
Constipation	28.204±20.141	31.413±30.551	43.594±32.701	2.972	0.054
Diarrhea	28.843±27.101	29.493±29.271	28.843±27.041	0.008	0.992
Financial impact	38.464±32.613	41.664±32.931	55.134±36.091^△※^	3.537	0.032
Global quality of life	58.637±22.761	53.194±15.598	45.810±14.351^△※^	6.693	0.002
If the scores are normal distribution, they are expressed as Mean±SD, and use analysis of variance, SNK comparative method; if the scores are not normal distribution, they are expressed as median, and use Kruskal-Wallis rank sum test; ^*^Compare with before chemotherapy and after 2 cycles, *P* < 0.05；△Compare with after 2 cycles and after 4 cycles, *P* < 0.05; ^※^Compare with before chemotherapy and after 4 cycles, *P* < 0.05.					

### 2.4 生活质量影响因素

女性较男性在疲倦及食欲减退症状得分高，症状重（*P* < 0.05）。文化程度低（受教育 < 9年）的患者较文化程度高（受教育 > 9年）的患者疲倦及失眠症状得分均高，文化程度低的患者疲倦及失眠症状重（*P* < 0.05）。按年龄将患者分为中年组（年龄 < 60岁）及老年组（年龄≥60岁），老年患者疲倦、恶心与呕吐症状、食欲减退症状均较中年人得分高，症状重（*P* < 0.05）。按性别、文化程度、年龄分别分组比较，组间在其他维度的比较无统计学差异（*P*>0.05）。按不同病理类型肺癌、Ⅲ与Ⅳ期、是否患基础病分别分组，患者的生活质量无统计学显著差异（*P*>0.05）。按BMI分消瘦、正常、超重三组，三组患者生活质量无统计学差异（*P*>0.05）。按患者血浆白蛋白水平将患者分为2组（低蛋白血症组白蛋白 < 35 g/L，正常白蛋白组白蛋白≥35 g/L）。两组比较，生活质量无统计学差异（*P*>0.05），但低白蛋白血症组躯体、角色、情绪、认知、社会、总健康得分均较正常白蛋白患者低，而疲倦、恶心及呕吐、疼痛、气促、失眠、厌食、便秘症状得分及经济困难得分较正常白蛋白患者高（表 2）。

**2:** 白蛋白水平对肺癌患者生活质量的影响（Mean±SD） Effect of albumin levels on the quality of life in patients with lung cancer (Mean±SD)

	Low albumin group	Normal albumin group	*t*	*P*
Physical	74.363±10.637	77.831±13.712	0.847	0.401
Role	64.864±25.691	65.126±23.861	0.036	0.971
Emotional	56.461±26.282	65.091±25.862	0.907	0.369
Cognitive	62.962±33.021	65.091±28.35	0.200	0.842
Social	53.691±24.713	65.113±22.976	1.342	0.186
Global quality of life	50.923±28.413	60.253±21.445	1.121	0.268
Fatigue	53.113±24.071	49.027±25.582	0.044	0.662
Nause and vomiting	24.039±22.211	21.06±17.582	0.497	0.621
Pain	25.912±18.412	25.59±18.412	0.035	0.972
Dyspnoea	49.601±34.423	48.160±33.814	0.115	0.909
Insomnia	37.031±31.146	31.001±25.201	0.516	0.608
Appetite loss	40.731±32.401	32.562±30.432	0.725	0.472
Constipation	40.748±36.391	25.589±20.971	1.087	0.282
Diarrhea	29.623±22.301	28.672±25.501	0.079	0.937
Financial impact	44.443±37.301	37.209±31.053	0.602	0.550

## 3 讨论

肺癌是常见的恶性肿瘤之一。大部分患者初诊时已失去手术机会，化疗是治疗肺癌的有效方法之一，但可能会产生一定的不良反应。在对肺癌患者治疗时，不仅要考虑治疗的生存时间、有效率等，还要考虑患者的生活质量。在治疗患者的过程中，不仅要关注和改善患者的躯体，还要关注和改善患者的心理和社会因素。

### 3.1 化疗前后生活质量的变化

本研究通过化疗对肺癌患者临床疗效及生活质量的评估，发现经过化疗部分患者肿瘤减小，临床症状缓解，化疗使患者临床获益。但部分患者生活质量较化疗前下降。随着化疗的进行，患者的呼吸困难有所改善，但患者情绪、角色、社会功能、恶心与呕吐、食欲减退、疼痛、经济困难恶化。患者本身患恶性疾病，且需承受化疗消化道反应、脱发、骨髓抑制等副作用，反复住院，每次住院都要花费一定的费用，不能保持正常的家庭、社交及工作等，往往情绪低落，有时存在焦虑、抑郁症状^[8]^。正如有的文献^[9]^报道癌症患者中抑郁的发生率高于正常人，严重影响患者的生活质量。有的患者想通过自杀结束生命以解除病痛的折磨。所以，我们要对肺癌患者给予高度关心，注重患者的心理等变化，及时给予患者疏导及干预治疗。

### 3.2 肺癌患者生活质量的影响因素

本研究发现女性肺癌患者较男性肺癌患者易倦怠、食欲减退。文化程度低者较文化程度高者易疲倦、失眠，我们分析认为文化程度越高，相对经济收入较好，对于医疗支出的担忧较少，能获得较多的医疗资源，因此生活质量较好。正如Ashing等^[10]^对703例乳腺癌患者进行调查，低收入、高压力的患者生活质量较差。本研究还发现老年患者较中年患者易疲倦、恶心、食欲减退，可能是肺癌患者年龄越大，身体功能越差、整体生活质量越低。很多肺癌患者存在营养不良，这与癌症本身治疗的副反应及肿瘤的恶性消耗有关。本研究按白蛋白水平进行分组，发现低蛋白血症患者的生活质量差。Sanchez-lara^[11]^对119例进展期非小细胞肺癌患者的患者营养状况、生活质量、生存期进行研究，发现营养不良患者的生活质量低，生存期较短。我们应注重患者的营养状况等躯体因素以提高患者的生活质量、生存期以及对治疗的耐受性。

总之，化疗能够改善部分肺癌患者部分临床症状，使部分肺癌患者临床获益，但随着化疗周期的增加，患者的躯体、角色、社会、情绪等逐渐变差，且易出现疲乏、食欲减退、恶心等症状，经济上逐渐出现困难。患者在治疗康复的过程中，身心均受到一定程度的影响，需要医务人员、亲友及社会多方面的关心支持。医务工作者要全面了解患者的情况，对其生活质量等予以评估，要把生活质量作为制定治疗方案考虑的主要因素，制定个体化的治疗方案，要特别注意化疗引起的不良反应并及时采取措施，告知患者及家属化疗中及出院后的注意事项，并给予患者正确、有效的心理疏导及有效的干预措施，注重改善患者营养状况，及时予营养支持，使肺癌患者受益。
